# Effect of alternate nostril breathing on cardio respiratory parameters and muscle strength among rotating shift workers

**DOI:** 10.6026/97320630017320

**Published:** 2021-02-28

**Authors:** Karthika Priyadharshini Udaykumar, Kavitha Ukkirapandian, Selvakumaran Selvaraj, Dhivya Kannan

**Affiliations:** 1 Sri Venkateshwara Medical College Hospital & Research Centre, Pondicherry, India; 2 Meenakshi Medical College Hospital & Research Institute, Kanchipuram, India; 3 Indira Gandhi Govt Medical College Hospital & Research Institute, Pondicherry, India

**Keywords:** Night shift workers, alternate nostril breathing, cardiorespiratory parameters

## Abstract

Night shifts at work is the most frequent reasons for circadian rhythm disruption and subsequent psychological and physiological disturbances, especially increased risk of cardiovascular and respiratory ailments compared to daytime workers. Alternate nostril breathing for about 15 minutes was known to have effect over cardiac, respiratory parameters and muscle strength. Hence aim is of interest to assess the effects of alternate nostril breathing (ANB) on cardio-respiratory parameters and muscle strength among the rotating shift workers in the tertiary care hospital. This observational study was carried out in the department of Physiology after getting institutional ethical committee clearance. Around 140 rotating night shift workers of both sex of age 25-40 years with normal BMI and 140 non-shift workers age, sex and BMI matched were selected as study and control group respectively. Heart rate, blood Pressure, respiratory rate, peak expiratory flow rate, respiratory endurance, respiratory burst test, muscle strength and fatigue were recorded before and after 15 minutes of ANB. Shift workers were found to have significantly altered systolic (P=0.000) and diastolic (P=0.002) blood pressure and heart rate (P=0.010) compared to non-shift workers. Fatigue is altered significantly (P< 0.05) after ANB between both shift and non- shift workers. ANB can be used as a therapeutic module among the shift workers, to maintain their sound health and to improve their performance in the night duty.

## Background

Shift work among the hospital staff is mandatory for providing round the clock care to the patients [1]. Night shift is the most common reason for disruption in the circadian rhythm, which results in internal de-synchronization and subsequent psychological and physiological disturbances among these workers [2]. Circadian rhythm controlled by supra- chiasmatic nucleus help us to co-ordinate our internal biology with the external environment and to adapt to light and dark cycle [3]. These workers suffer from persistent fatigue and decreased muscle strength and reduced job performance [4,5]. Norepinephrine was found to be elevated abnormally among shift workers [6]. Lung function is affected in these types of workers [7]. Yoga is a system of philosophy established in India thousands of years ago. Pranayamas are breathing techniques that exert profound physiological effects on pulmonary, cardiovascular and mental functions [8]. These techniques are gaining more importance and becoming acceptable to the public as well as scientific community [9]. According to yoga, ANB is a phenomenon of the alteration in the flow of subtle energy in the IDA and PINGALA. This fact was incorporated in development of pranayama, a technique of controlled breathing [10]. ANB is a simple technique, which was known to alter blood pressure, heart rate, respiratory rate, PEFR, muscle strength on acute exposure for about 15 minutes [9, 11,12]. Hence it is of interest to assess the effects of alternate nostril breathing (ANB) on cardiorespiratory parameters and muscle strength among the rotating shift workers in the tertiary care hospital.

## Methodology

This cross-sectional study was done in a medical college in Sri Venkateshwaraa Medical college hospital and research center, from the month of April to July 2018. Group I includes 140 volunteers of both the genders of age 25-40 years who did rotating night shifts at least for past 6 months and Group II includes 140 volunteers of both the genders of age 25-40 years who did not do night shifts in the last two years. Group I consists of 66 male, 74 female nursing staffs, nursing assistants and technicians. Group II consists of 72 male, 68 female nursing staffs, nursing assistants and technicians. BMI between 18.5 to 22.9 kg/m2 in both the groups was included by simple random sampling method (Table 1). Subjects with known history of diabetes mellitus, hypertension and hormonal abnormalities like hypo or hyperthyroidism, pregnant women, asthmatics; those who are on chronic medication, athletics, and yoga practitioners were excluded from the study. Written informed consent was obtained from all the participants after getting Institution ethical committee clearance. All the subjects were assessed in the research lab of Physiology department from 10 -12 am. After 10 minutes rest, procedures were explained to the participants and cardiac parameters like blood pressure (using sphygmomanometer) and heart rate was recorded in sitting posture. Following respiratory parameters were recorded [13,14].

## Respiratory efficiency tests [13]:

1. Peak Expiratory Flow Rate (PEFR): After a deep inspiration subject was asked to expire forcefully into the mouth piece of the wright's Peak flow meter after adjusting the knob to zero level. Three successive trails were performed and the maximum value was recorded.

2. Expiratory blast test: The rubber tube of sphygmomanometer was disconnected from the mercury reservoir to the cuff. The participants were instructed take deep inspiration and to expire to the maximum into the mercury manometer of a sphygmomanometer to raise the mercury level as high level as possible. Three successive trials were performed and the maximum value was recorded.

3. Respiratory endurance tests: Participants were instructed to take maximum inspiration and to expire into the mercury manometer of a sphygmomanometer and raise the level up to 40mmHg and to maintain it as long as possible. Three successive trials were performed and the maximum value was recorded. The time (in seconds) that the participant could maintain the mercury level at 40mmHg was recorded.

Nose clip was applied while expiration in PEFR, expiratory blast test and respiratory endurance test.

## Muscle strength and fatigue (hand grip endurance) [12]:

To test the muscle strength and fatigue the subjects were instructed to sit comfortably. They were asked to perform maximum voluntary contraction (MVC) using the handgrip dynamometer. The test was repeated three times with a gap of two minutes and the highest value was recorded as handgrip strength (HGS). Following HGS, the subjects were instructed to maintain one-third of HGS as long as possible. Duration in seconds was noted as hand grip endurance (HGE) using the stopwatch which indicates fatigability of the muscle. ANB was practiced between both the groups for 15 minutes and all the parameters were recorded again immediately after ANB.

## Alternate nostril breathing:

The subjects were asked to take a steady sitting posture comfortably with the head, neck and trunk erect, in a straight line in our yoga lab. The subject was instructed to fold the index and middle fingers of the right hand so that the right thumb can close the right nostril and the ring finger can close the left nostril (Vishnu Mudra). With the right nostril closed by the right thumb, exhale completely through the left nostril. The exhalation should be, controlled and free from exertion and jerkiness. At the end of the exhalation the subject was asked to close the left nostril with the ring finger, open the right nostril and inhale slowly and completely. Inhalation should be smooth, controlled and of the same duration as exhalation. Repeat this cycle twice. Now exhale completely through the right nostril keeping the left nostril closed with ring finger. In summary, one exercise consisted of 3 cycles of exhalation through the left nostril and inhalation through the right nostril followed by 3 cycles of exhalation through the right nostril and inhalation through the left nostril and this was repeated for about 15 min [10]. Statistical Analysis: All the data were tabulated as mean ± SD. Statistical analysis was done using SPSS 16. Students unpaired t tests was done to compare the parameters between the groups. Students paired t test was done to compare all the parameters before and after the ANB among the groups.

## Results and Discussion:

Table 1 shows that both the groups are found to be age and BMI matched and hence they are comparable. Table 2 shows that SBP and DBP are significantly higher among the study group compared to the control group, which could be attributed to the sympathetic enhancement. It has been reported that disrupted circadian rhythm due to shift work leads to hypertension, dyslipidemia, diabetes and obesity. [14] Shift workers had significantly increased vascular and cardiac stress even during the next day off after the night shifts.[15] Thus the continued alteration of circadian cycle among the rotating shift workers may lead to the development of hypertension and cardiovascular disease in the long run due to altered melatonin secretion involved in oxidative stress. [16,17] Thus increased blood pressure in shift workers might be due to enhanced sympathetic activity and lower heart rate might be due to baroreceptor reflex compensatory mechanism. Higher heart rate among the non-shift workers could be due to duty related stress as the participation timings were during their duty hours unlike study group. Respiratory endurance and PEFR were non-significantly decreased among the shift workers similar to the study done by Arti et al. where PEFR is decreased among elderly shift workers. [7] The handgrip endurance shows that shift workers undergo easy fatigability. Similarly, Vallieres et al. reported fatigue among the individuals with insomnia. [18] Irtyah et al. reported better muscle strength and PEFR among shift workers who worked less than 10 years. [5]

Table 3 and Table 4 show that SBP, DBP and HR decreased after ANB in both the study and control group. Various authors have also reported decreased HR, SBP in healthy, decreased SBP, and DBP in hypertensive after ANB. [9,19] Yoga has conditioning effect on autonomic function mediated through the limbic system and higher centers in CNS.[20] The respiratory rate is also decreased significantly after ANB. The neural reflex mechanism in the superior nasal meatus during ANB might be the reason for parasympathetic dominance hence decreased respiratory rate.[21] ANB removes their concentration from the worldly worries and de-stress them. It decreases adrenaline release and thereby decreasing the heart rate, blood pressure and respiratory rate. [22] During lung inflation, stimulated pulmonary stretch receptors bring about withdrawal of sympathetic tone in the skeletal muscle blood vessels increases vasodilation and decreases DBP.[23] Vasodilatation might also have increased the oxygen supply to the skeletal muscle resulting in decreased muscle fatigue and increased the muscle endurance. Increased HGS following ANB might be due to improved autonomic tone and hypo-metabolic state, which leads to increase the concentration on the task. [12] Decrease in elastic and viscous resistance of the lungs during inspiration increases respiratory muscle efficiency after ANB. It is known to increase the compliance by releasing surfactant and prostaglandins. [24,25] Cleansing the airway, efficient use of the diaphragm and abdominal muscles during the practice of ANB raise PEFR. Pranayama decreases constrictor tone of the smooth muscles of respiration. [26] Deep inspiration stimulates stretch receptors that relaxes the smooth muscles of larynx and tracheobronchial tree, modulates the airway caliber with reduction in the airway resistance. [27] Anandha et al. have also reported immediate alteration in cardiopulmonary responses after ANB.[28] Table 5 shows that percentage decrease in SBP and increase in RB, PEFR and HGS after ANB compared to the basal value were higher among the shift workers. Percentage decrease in DBP, HR, RR and increase in HE after ANB shows a greater change among the non-shift workers. Thus long term practice of ANB might be needed to bring greater change in these parameters among shift workers.

## Conclusion

ANB has an immediate effect on cardiorespiratory parameters, muscle strength and endurance among the shift and non-shift workers. It can help to revert the alterations in various physiological parameters resulted from the disturbed circadian rhythm. It also improves performance and level of job satisfaction.

## Figures and Tables

**Table 1:** Age and BMI variations between the groups

	Group I	Group II	P value
Age (years)	30.94±4.251	30.03±4.269	0.0724
BMI (Kg/m2)	21.93±1.930	21.73±1.928	0.3831

**Table 2:** Basal cardiorespiratory parameters, muscle strength and fatigue before ANB

Parameters	Group I	Group II	P value
SBP (mm Hg)	113.21±4.742	110±6.92	0.000*
DBP (mm Hg)	75.56±10.28	72±6.49	0.002*
HR (beats/ min)	80.11±11.63	83.46±9.71	0.010*
RB (mm Hg)	58.24±10.91	58.71±14.35	0.176
RE (secs)	25.83±11.13	27.36±11.27	0.674
RR (breaths/min)	15.21±2.92	15.41±2.98	0.761
PEFR (L/min)	295.26±118.38	316.93±123.31	0.255
MS/HGS (kg)	23.83±8.15	23.38±9.63	0.585
HGE/ fatigue (secs)	127.57±20.46	131.15±23.53	0.135

**Table 3:** Effect of alternate nostril breathing exercise on cardio-respiratory parameters, muscle strength and fatigue among Group I

Parameters	Before ANB	After ANB	P value
SBP (mm Hg)	113.21±4.74	108.73±6.69	0.000*
DBP (mm Hg)	75.56±10.28	72.07±9.46	0.000*
HR (beats/ min)	80.11±11.63	77.66±7.76	0.001*
RB (mm Hg)	58.24±10.91	63.84±10.87	0.000*
RE (secs)	25.83±11.13	31.81±12.69	0.000*
RR (breaths/min)	15.21±2.92	14.45±2.61	0.003*
PEFR (L/min)	295.26±118.38	338.70±117.34	0.000*
MS/HGS (kg)	23.83±8.16	27.34±8.49	0.000*
HGE (secs)	127.57±20.46	128.65±19.23	0.015*

**Table 4:** Effect of alternate nostril breathing exercise on cardio-respiratory parameters, muscle strength and fatigue among Group II

Parameters	Before ANB	After ANB	P value
SBP (mm Hg)	110±6.92	106.41±6.66	0.000*
DBP (mm Hg)	72.30±6.48	67.84±6.24	0.000*
HR (beats/ min)	83.46±9.70	78.31±8.29	0.000*
RB (mm Hg)	58.71±14.36	63.82±15.46	0.000*
RE (secs)	27.36±11.27	32.44±12.48	0.000*
RR (breaths/min)	15.41±2.99	14.37±2.537	0.000*
PEFR (L/min)	316.93±123.311	357.93±120.76	0.000*
MS/HGS (kg)	23.38±9.639	24.09±9.82	0.012*
HGE/fatigue (secs)	131.15±23.53	133.14±22.17	0.000*

**Table 5:** Percentage change in cardiorespiratory parameters, muscle strength and fatigue before and after ANB among Group I & Group II

Parameters	Group I	Group II
% Decrease in SBP	3.95%	3.26%
% Decrease in DBP	4.61%	5.70%
% Decrease in HR	3.05%	6.17%
% Increase in RB	9.61%	8.70%
% Increase in RE	23%	18.50%
% Decrease in RR	4.90%	6.70%
% Increase in PEFR	14.71%	12.70%
% Increase in MS/HGS	14.72%	3.03%
% Increase in HGE/ fatigue	0.84%	1.51%
